# Community assembly of coral reef fishes along the Melanesian biodiversity gradient

**DOI:** 10.1371/journal.pone.0186123

**Published:** 2017-10-25

**Authors:** Joshua A. Drew, Kathryn L. Amatangelo

**Affiliations:** 1 Department of Ecology, Evolution and Environmental Biology, Columbia University, New York, NY, United States of America; 2 Division of Vertebrate Zoology, American Museum of Natural History, New York, NY, United States of America; 3 Department of Environmental Science and Biology, The College at Brockport, State University of New York, Brockport, NY, United States of America; Leibniz Centre for Tropical Marine Research, GERMANY

## Abstract

The Indo-Pacific is home to Earth’s most biodiverse coral reefs. Diversity on these reefs decreases from the Coral Triangle east through the islands of Melanesia. Despite this pattern having been identified during the early 20^th^ century, our knowledge about the interaction between pattern and process remains incomplete. To evaluate the structure of coral reef fish communities across Melanesia, we obtained distributional records for 396 reef fish species in five taxa across seven countries. We used hierarchical clustering, nestedness, and multiple linear regression analyses to evaluate the community structure. We also compiled data on life history traits (pelagic larval duration, body size and schooling behavior) to help elucidate the ecological mechanisms behind community structure. Species richness for these taxa along the gradient was significantly related to longitude but not habitat area. Communities are significantly nested, indicating that species-poor communities are largely composed of subsets of the species found on species rich reefs. These trends are robust across taxonomic groups except for the Pomacentridae, which exhibit an anti-nested pattern, perhaps due to a large number of endemic species. Correlations between life history traits and the number of reefs on which species occurred indicate that dispersal and survival ability contribute to determining community structure. We conclude that distance from the Coral Triangle dominates community structure in reef fish; however, conservation of the most species-rich areas will not be sufficient alone to conserve the vivid splendor of this region.

## Introduction

Species richness gradients are a ubiquitous feature of the earth’s biota. While the most well-known of these is the latitudinal gradient in species richness [1,2], other species gradients, including longitudinal [3] and elevational [4] are also present. These gradients exemplify the heterogeneous distribution of biodiversity on earth and inform the causative mechanisms underlying its maintenance. Understanding the causes and consequences of diversity gradients is critical in a time of global change, when limited resources must be deployed to conserve as much diversity as possible. Conservation efforts are often focused on target species or groups, and understanding community composition is equally, if not more, critical to conservation than richness numbers alone as we transition away from species based management and towards ecosystem based approaches to conservation. Understanding the predictability of community composition based on characteristics including dated phylogenies and their relationship to earth history is an area of active research [5–7].

The Indo-West Pacific is the most diverse shallow water marine environment on Earth. Within this area of diversity, the Coral Triangle, bounded by Indonesia, the Philippines, and Papua New Guinea, represents the epicenter of marine biodiversity for a variety of taxa ranging from fish to mangroves [8,9]. Three major theories have been postulated to explain why that region contains such a rich fauna. The Center of Overlap postulates that the Coral Triangle has an elevated species richness because it lies at an ecotone between the Indian and Pacific oceans and thus draws from both species pools [10–12]. The Center of Accumulation reflects upon the generalized patterns of oceanic movement, drawing from the Pacific into the Coral Triangle, suggesting that species will be flushed into the Coral Triangle [13,14], while the Center of Origin draws upon the region’s tumultuous geological past and dynamic rise and fall of oceans to create vicariant events which would allow for rapid rates of speciation [15,16]. Furthermore, a non-exclusive Center of Survival theory suggests that extinction rates are reduced within the Coral Triangle allowing for the maintenance of high species diversity [7,17]. While each of these theories has empirical evidence to support it, ascribing a single overarching theory is perhaps simplistic. Rather, a multi-theory, pluralistic approach may best explain the high diversity in the Coral Triangle, and by extension the western parts of Melanesia [18].

Diversity in the Indo-West Pacific is not homogenous, but occurs along a longitudinal gradient such that as one moves east from the Coral Triangle towards Samoa there is a sharp decline in the number of species. Although this gradient was first described in the early 20^th^ century, the mechanisms underlying this pattern are not readily apparent as there are few obvious allopatric barriers to dispersal for marine organisms [19,20]. Moreover, while the pattern has been described at the gross level, the relative contribution of individual taxonomic groups towards that diminution has not been fully explored, and could be important for delineating areas of endemism. Additionally, the relationship this radial loss of diversity has on the composition of local reef communities has not been quantified.

**Fig 1:**
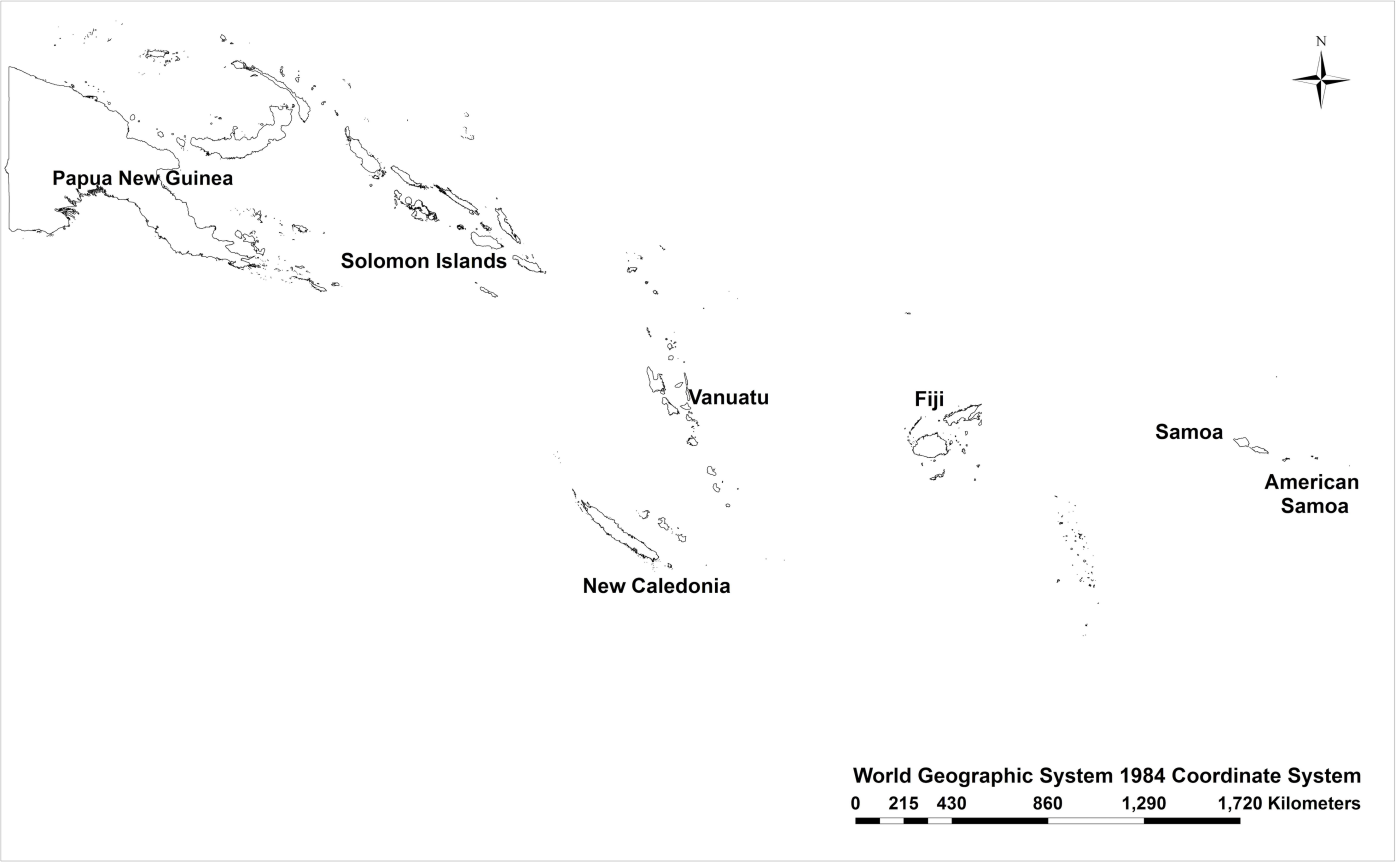
Map of Melanesia showing major archipelagos sampled in this study. Map made with ArcMap v10.3.1.

**Fig 2:**
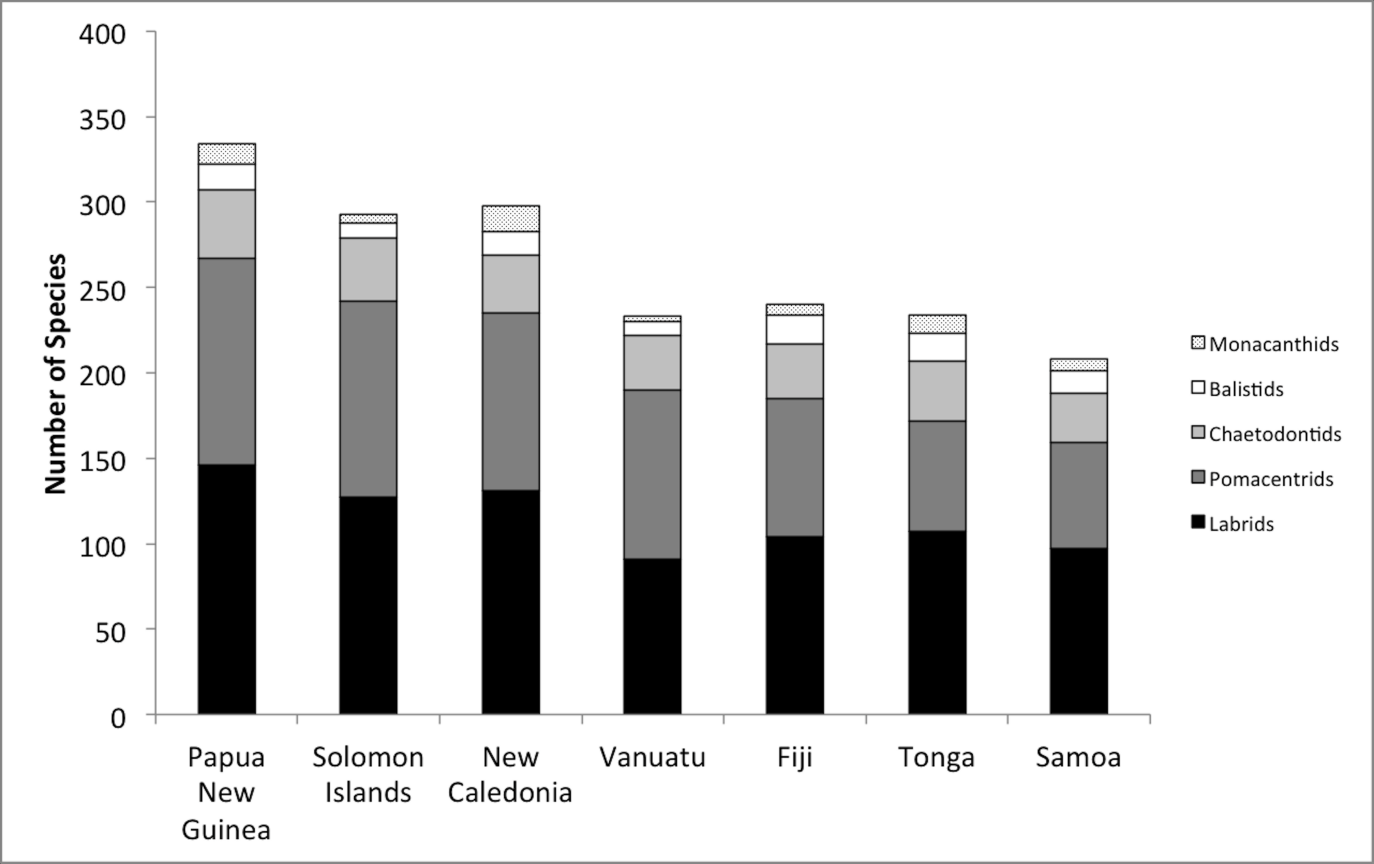
Relative species richness of each of five coral reef fish taxa reported from seven countries across Melanesia. Countries are ordered from west to east along the x-axis, with Papua New Guinea being the westernmost site.

The islands of Melanesia (from west to east), Papua New Guinea, The Solomon Islands, New Caledonia, Vanuatu, Fiji, Tonga and Samoa (including American Samoa), span approximately 44 degrees of longitude and contain over 35,000 km^2^ of reef area, with a general trend of greater habitat availability in the more species-rich west than in the smaller, and less diverse, archipelagos of the east (Fig 1, Fig 2, Table 1). The reefs within this region are largely oceanic in origin and are fairly evenly distributed with the notable exception of an 800-kilometer stretch of deep water overlaying the Fiji Basin, which lies between Vanuatu and Fiji (Fig 1). This approximately 12 million-year-old gap [21] is deep and contains no extant shallow water habitats for adult coral reef organisms. Stepping-stone islands are important in the colonization of islands in Melanesia [22,23] because they provide intermediate habitat for species, reducing but not eliminating the impacts of dispersal limitation on communities [24]. The lack of habitat between the eastern and western habitat has been important in structuring terrestrial arthropod communities [25] and may also play a role in aquatic ecosystems.

**Table 1:** Reef area (km), longitude, number of species, and mean (stddev) occurrences of species in seven coral reef taxa reported from seven archipelagos across Melanesia.

Locale	Reef Area (km)	Longitude	Number of species	Occurrences
Papua New Guinea	14,536	148° 45.931'E	334	5.08 (1.84)
Solomon Islands	6,744	159° 26.848'E	293	5.29 (1.76)
New Caledonia	7,450	165° 24.524'E	298	5.32 (1.79)
Vanuatu	4,110	168° 20.333'E	233	5.77 (1.48)
Fiji	6,704	178° 43.517'E	240	5.77 (1.54)
Tonga	1,500	174° 34.339'W	234	5.83 (1.47)
Samoa	786	172° 8.280'W	208	6.12 (1.26)

The ability of a species to disperse among countries is mediated through individual species traits. In order to colonize a series of reef habitats propagules must first be able to reach adjacent reef, followed by survival and establishment of viable populations. Traits of particular relevance for species’ dispersal ability are those influencing individual dispersal capability (larval duration) and the mitigating post-settlement mortality (schooling behavior, body size). For example, physical and behavioral traits such as body size, schooling and propensity to raft have all been found to correlate with range size due to their influence on larval success [26–28]. Any trait that influences the success of a species’ larvae may also affect the connectivity of populations and patterns of community composition among habitats, although the relationship between larval duration and genetic connectivity is equivocal [29–31]. Using traits to understand potential mechanisms behind macroecological patterns like the Melanesian diversity gradient can further assist efforts for conservation in the region by shedding light on what factors may be most at risk (e.g. loss of habitat, disruption of spawning aggregations etc.).

Understanding how the Melanesian diversity gradient intersects with patterns of endemism and community composition is important for prioritization of regional conservation. Although biogeographic information is routinely incorporated in conservation planning [32], much of this has been limited to single species or small sets of species, often with idiosyncratic results [33]. Applying more nuanced biogeographic analyses of species diversity data offers the potential to identify the proper biogeographic scale for which to draft regional management plans as well as aiding in the prioritization of conservation actions [34,35]. In particular, it is important to know whether each reef has its own ecologically independent community, or whether the reef communities in countries are nested so that western countries (closer to the Coral Triangle) serve as source communities for relatively depauperate eastern reefs in Melanesia (Center of Origin theory [36]). If such nestedness is found, then conservation of species-rich western reefs may also provide protection for species who are constituents of lower diversity eastern reef communities.

In this study we use distributional records and life history traits to quantify and explain community structure across the Melanesian biodiversity gradient. We specifically tested the hypotheses that:

1. Species-poor eastern communities are a subset of species-rich western communities closer to the Coral Triangle, affecting both community structure and species richness.
2. The approximately 800 km gap of open ocean between Vanuatu and Fiji which overlies the Fiji plateau results in disjunct community structure among Melanesian coral reef fish communities.
3. Life history traits that correlate with greater dispersal ability (pelagic larval duration) and improved post-settlement survival (body size, schooling behavior) will explain the fidelity of species to community-scale patterns of nestedness and richness.

## Methods

### Data collection

We obtained distributional records for 396 reef fish species in the families Balistidae, Chaetodontidae, Labridae (including Scarine Labrids), Monacanthidae, and Pomacentridae across seven sites in Melanesia (Table 1, S3 Table). We chose these taxonomic groups because they are distributed across the Actinopterygiian tree of fishes [37], they cover a wide range of life history traits, and they are the most speciose groups that are both readily identifiable and highly representative of Melanesian reef fish fauna. We generated a presence-absence matrix across these those locales using data from museum collections records accessed through FishNet (www.fishnet2.net), peer reviewed literature including fish check lists, and biological inventories (S2 Table). All data were evaluated through the records of The California Academy of Sciences’ Catalog of Fishes (www.calacademy.org/scientists/projects/catalog-of-fishes) and The Encyclopedia of Life (www.eol.org) to ensure that all species adhere to their current taxonomic designation. We also double checked data against the publically available database FishBase (www.fishbase.org). Public bioinformatic databases, including FishBase, are known to have varying levels of quality controls which can introduce errors into their analyses [38]. Therefore, we relied on mulitple instances of reference (both museum collections, or multiple peer reviewed literature) before including species in our dataset.

We focused our research longitudinally across seven archipelagos from west to east: Papua New Guinea, The Solomon Islands, New Caledonia, Vanuatu, Fiji, Tonga, and Samoa (including American Samoa). To avoid misidentification, we exluded Caribbean or Indian Ocean species reported from one locale in Melanesia that were unknown elsewhere in the Indo-Pacific.

We calculated the shortest straight-line distance between each pair of islands, and identified the maximum geographic extent of each species in km. For each species we also gathered trait data on schooling behavior (yes/no) and body size (standard length, measured in centimeters). We also compiled pelagic larval duration data for 189 species of fish, which included representatives from all five families (S3 Table). Trait data were gathered from peer reviewed literature with supplemental information from Fishbase (S4 Table).

### Statistical analyses

We used hierarchical clustering methods to determine how reef fish communities are structured among countries We clustered reef fish communities using the function ‘hclust’ in the R package vegan based on Jaccard distances [39, 40]. We used the ‘average’ clustering algorithm, which calculates distances based on the average dissimilarity between groups, as it is more robust than minimum or maximum distance methods. In order to quantify the number of significantly different clusters in our dendrogram we used the function ‘simprof’ in the R package clustsig to test the null hypothesis that there is no group structure [41]. Moving from the top level of the dendrogram, we tested clustering structure between each subsequent split down the tree continuing only if the testing result was significant. The significant clusters found by this testing procedure are the groups of islands with the most similar reef fish communities. We set significance levels to 0.01 because the procedure involves multiple tests.

Beta diversity, the amount of species turnover between two different sampled areas, can be divided into two constituent components, richness and replacement [42]. Differences between two countries could come from differences in richness (also called nestedness), wherein the change in biodiversity is due to a winnowing away from a common pool, or through replacement/turnover, wherein the differences between countries is due to the countries’ reefs drawing from different constituent faunas. We evaluated the relative contribution of richness and replacement by calculating the components of beta diversity. We used two different partitioning frameworks [43,44], using the ‘BAT’ and ‘betapart’ packages for R [42,45–47].

We quantified the relationships among reef area, species richness, and longitude using correlation analysis and a multiple linear regression model. We calculated the relative importance of reef area and longitude with the lmg metric using the function calc.relimp in the R-package relaimpo [48]. The lmg metric accounts for potential dependencies between regressors to make comparisons of relative importance.

To evaluate how fish species found at lower diversity countries in the east compare to those found at higher diversity countries towards the west we performed nestedness analyses [49]. Nestedness analysis determines if lower diversity countries contain non-random subsets of species from higher diversity countries. Non-random subsets can be nested (species at lower diversity countries are subsets of those found at high diversity countries) or anti-nested (species at depauperate countries are non-random sets of species not necessarily found at more species rich countries). Anti-nestedness may result from turnover of species along gradients or so called checkerboard patterns [50]. We used the FORTRAN95 software NODF-Program to implement the NODF (Nested metric based on Overlap and Decreasing Fill) nestedness metric [51]. NODF calculates both overall nestedness and the contribution of countries (columns) and species (rows) to the nestedness patterns. We used a ‘fixed-fixed’ null model for randomization of species presences that preserves row and column totals, which have low Type 1 error rates and high statistical power [49,52]. We selected a ‘proportional’ null model and created 1000 random matrices for each run by performing swaps equal to ten times the matrix size. We ran each model twice, once using the default settings that order countries (columns) by species richness, and additionally by ordering the countries by longitude. This allowed us to directly test whether nestedness is occurring along the longitudinal gradient, regardless of differences in species richness. Nestedness analyses with countries ordered by longitude were performed for both schooling and non-school fish to evaluate whether schooling behavior affected community structure.

We evaluated the impact of traits on geographic extent using two linear mixed-effect models with geographic extent as the response variable. One model, for all species, included schooling and body size as fixed effects. The other model, for the subset of species for which we compiled larval duration, also included larval duration as a fixed effect. Both models factored out phylogeny with genus nested within family as a random factor. Analyses were performed on log-transformed data in JMP 12.1.0 [53].

## Results

Sampling of reef fish (5 family-level groups, 91 genera, 396 species) from seven countries across Melanesia resulted in a clear gradient of decreasing biodiversity moving from Papua New Guinea to the Samoan archipelago (Table 1). The Pomacentridae, Labridae (including Scarids), and Chaetodontidae are the most speciose groups, followed by the relatively species-poor Balistidae and Monacanthidae. The two most species-rich groups, Pomacentridae and Labridae, accounted for 81.1% of the total number of identified species (Fig 2). There is little taxonomic variation in this pattern of diminution; the loss in species numbers comes from a proportional loss of the species pool, not from the absence of any one particular family (Fig 2).

The majority of fish species are distributed across multiple countries (Table 1, mean number of countries occupied 4.65±2.04). Of 396 species in the dataset, over one quarter (28%) can be found at all seven countries. Twenty-eight (7%) are found only at one site. Fourteen of those single site species are found on New Caledonia, eight on Papua New Guinea, and three or less at each of the other countries.

When we look at the distribution of all 396 species as they span across the Fiji Plateau we find that 279 of all species (70.4%) are present on both sides. Moreover, 104 species (26.2%) are found only west of the trench in Papua New Guinea, the Solomon Islands, Vanuatu and New Caledonia, while only 13 species (3.2%) are found only east of the trench in Fiji, Tonga and Samoa.

**Fig 3:**
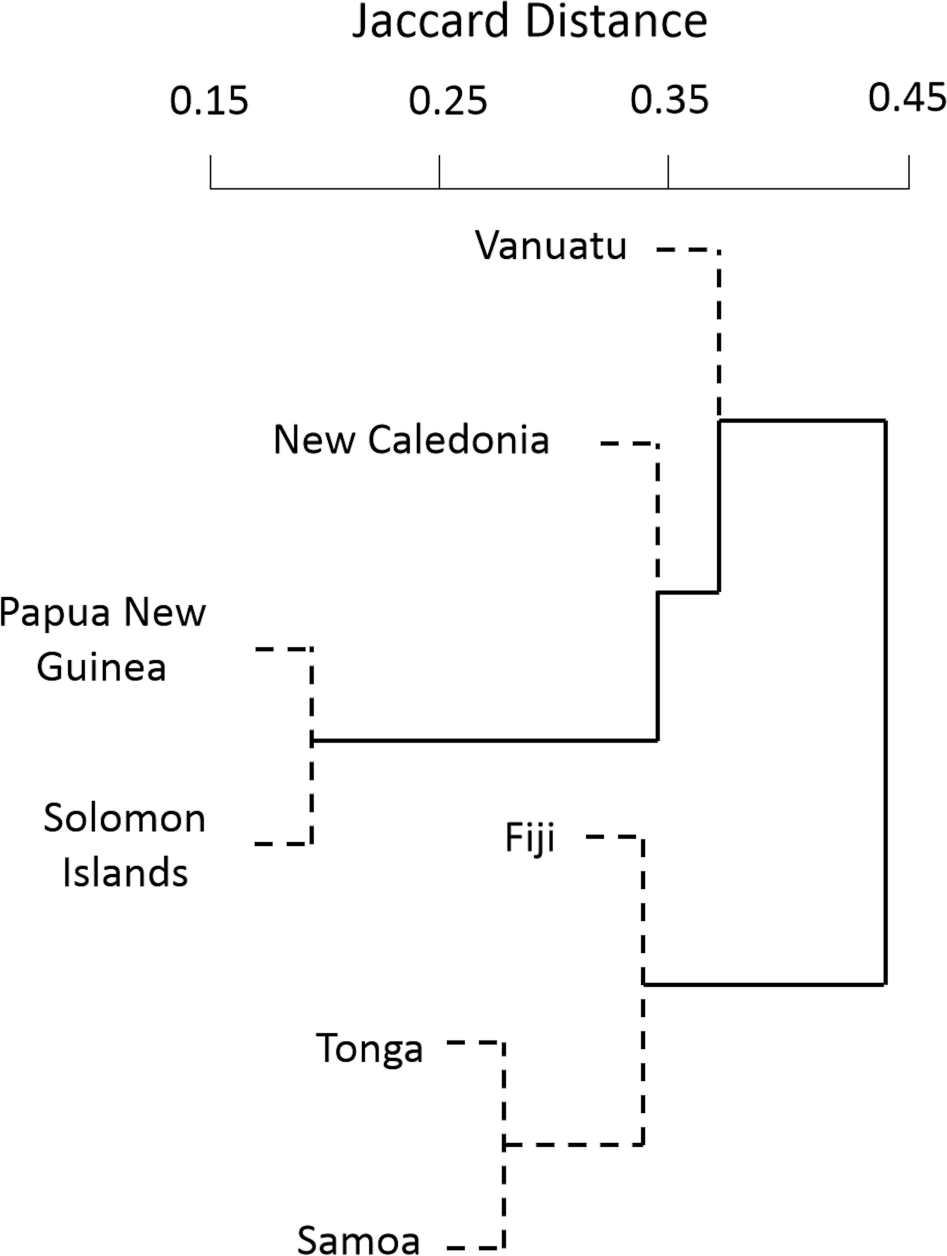
Dendrogram of reef fish communities for five taxa (Balistidae, Chaetodontidae, Labridae, Monacanthidae, Pomacentridae) built on Jaccard distances and clustered using group averages. Clusters significant at p<0.01 are indicated with solid lines. All clusters are significant at p<0.05.

The largest Jaccard distances were observed occurring between western Melanesia (Papua New Guinea, Solomon Islands, Vanuatu, New Caledonia) and the countries to the east of the Fiji Plateau (Fiji, Tonga, Samoa—Fig 3). The replacement/turnover component (representing different species pools) contributes from 22–98% of the beta diversity represented by Jaccard dissimilarities among these countries (Table 2). The replacement component was particularly large (>75% of beta diversity) between Vanuatu and each of Fiji, Tonga, and Samoa, between Fiji and Tonga, and between the Solomon Islands and New Caledonia. Cluster dendrograms based on total Jaccard distance, the replacement component, and the richness component were similar, although the middle islands (New Caledonia and Vanuatu) alternated depending on the metric (S1 Fig), perhaps driven by New Caledonia’s relatively high number of endemic species influencing the replacement component.

**Table 2:** Jaccard distance based on species presence-absences in reef communities across seven Melanesian countries. The first number in parentheses indicates the replacement (turnover) component of Jaccard distance following Carvalho et al. 2011, the second the turnover component following Baselga 2010.

	Papua New Guinea	Solomon Islands	New Caledonia	Vanuatu	Fiji	Tonga
**Solomon Islands**	0.193 (0.07, 0.08)					
**New Caledonia**	0.315 (0.22, 0.24)	0.376 (0.36, 0.37)				
**Vanuatu**	0.371 (0.08, 0.11)	0.366 (0.18, 0.22)	0.372 (0.17, 0.21)			
**Fiji**	0.419 (0.16, 0.22)	0.446 (0.29, 0.34)	0.384 (0.21, 0.25)	0.429 (0.41, 0.42)		
**Tonga**	0.435 (0.16, 0.22)	0.477 (0.31, 0.37)	0.363 (0.17, 0.21)	0.479 (0.46, 0.48)	0.313 (0.29, 0.30)	
**Samoa**	0.451 (0.09, 0.14)	0.458 (0.20, 0.27)	0.419 (0.14, 0.19)	0.479 (0.39, 0.43)	0.365 (0.25, 0.28)	0.280 (0.18, 0.20)

Correlation analyses demonstrated that species richness was significantly related to longitude (r = 0.87, p = 0.012) but not area (r = 0.73, p = 0.062). Area and longitude were significantly correlated (r = 0.89, p = 0.007). A multiple regression including both area and longitude has a R^2^ of 0.757 (p = 0.059). The results from the relative importance metric lmg indicate that longitude explains 48.5% of the total 75.7% variance explained by the model.

When all taxa were included in nestedness analyses, countries demonstrated significant nestedness when ordered by longitude but not by richness (Table 3). Overall nestedness NODF metrics and the ‘rows’ component (species) were not significant. When taxa were analyzed separately, the Pomacentridae were significantly anti-nested by all three metrics when ordered by either longitude or richness (S1 Table, S2 Table). Labridae and Balistidae had significant column (countries) nestedness when ordered by longitude. Non-schooling fishes were significantly nested when ordered by longitude, while schooling fishes were not (Table 4).

**Table 3:** Results of nestedness analyses for all species. A metric of 100 indicates perfect nestedness. NODF stands for “Nested metric based on Overlap and Decreasing Fill” for the whole data set, NODF columns focuses on individual countries, and NODF rows focuses on species.

	NODF			NODF columns			NODF rows		
	Metric	Z value	Pz(H0)	Metric	Z value	Pz(H0)	Metric	Z value	Pz(H0)
Ordered by Richness	73.09	-0.45	0.325	84.34	0.57	0.284	73.09	-0.41	0.342
Ordered by Longitude	73.09	-0.44	0.329	**73.87**	**4.31**	**<0.0001**	73.09	-0.41	0.342

Bold values have p<0.05.

**Table 4:** Results of nestedness analyses for species split based on schooling behavior. Islands were ordered longitudinally. A metric of 100 indicates perfect nestedness. NODF (Nested metric based on Overlap and Decreasing Fill) for the whole data set, NODF columns focuses on individual countries, and NODF rows focuses on species.

	NODF			NODF columns			NODF rows		
	Metric	Z value	Pz(H0)	Metric	Z value	Pz(H0)	Metric	Z value	Pz(H0)
Schooling (213)	73.63	-0.27	0.395	84.22	-1.51	0.065	73.62	-0.22	0.411
Not Schooling (183)	73.20	-0.53	0.299	**63.55**	**3.26**	**0.0005**	73.21	-0.47	0.319

Bold values have p<0.05.

A linear mixed-model for all species demonstrated that body size was a significant (p < 0.05) positive predictor of geographic extent (Table 5). On a subset of 173 of the species, pelagic larval duration was a significant (p < 0.05) positive predictor of geographic extent. Body size approached significance in that model (p = 0.0522). Schooling behavior was not statistically significant in either model.

**Table 5:** Parameter estimates from linear mixed-effect models predicting log geographic extent in km. Phylogenetic relatedness was factored out using random effects. Two models were evaluated, as larval duration data were available for only a subset of the fish taxa.

	Source	Estimate	Std Error	p
N = 173	Intercept	6.937	0.353	<0.0001*
	Log body size	0.072	0.069	0.2968
	Log larval duration	0.210	0.107	0.0522†
	Schooling	-0.017	0.046	0.7041
N = 342	Intercept	7.392	0.159	<0.0001*
	Log body size	0.141	0.053	0.0084*
	Schooling	-0.006	0.036	0.8747

* p < 0.05

† < 0.10.

## Discussion

The Coral Triangle represents the epicenter of tropical marine biodiversity and our results show that the diminution of species richness away from that center is both structured and taxonomically broad across coral reef fishes. Our results place a quantitative framework around a qualitative statement first made about the region’s fishes in 1906, when Jordan et al. [54] noted “*The fish fauna of Upolu and Tutuila* [Samoa] *is entirely the same*, *nor is there evidence of any divergence from the fauna of Tahiti*, *Tonga*, *and other islands of similar character*. *It is largely identical with that of the East Indies*, *from which nearly all of its elements are clearly derived*. *But a number of East Indian species fail to extend their range thus far to the east*, *very many of them not ranging- beyond the island of Papua or New Guinea*.” Thus, the first description of the Melanesian species diversity gradient invoked the longitudinal nestedness of those communities within Melanesia. This taxonomically broad pattern further went on to fuel such insights as Taxon Cycling [21, 55] and the Theory of Island Biogeography [56], and it is through this lens that we can continue to gain insight into the distribution of life in the region. The Island Theory of Biogeography predicts that species assemblages will be a function of extinction and colonization processes. However the classic MacArthur Wilson presentation of the theory treats all species equally, and while the overall predictions about the *number* of species may be supported empirically, work on tropical coral reefs and other systems suggest that the *kinds* of species experience these processes differentially, with for instance, higher trophic level guilds experiencing greater levels of extinction [57]. Species diversity across scales is clearly the result of a multitude of complex factors, which underscores the importance of incorporating phylogeny, species ecology and earth history when interpreting modern species distributions. Given that view, we examined the differential patterns of community assembly from both a species specific and functional trait perspective.

### Patterns of nestedness

Our results show that the reef communities in the countries in eastern Melanesia are significantly nested, and therefore contain a subset of the fish communities found in western Melanesia. Nestedness is more prevalent in reef communities when countries are ordered longitudinally than by richness, supporting the importance of distance away from the Coral Triangle to community composition. This west-to-east pattern of nestedness has also been recognized in this region for other fauna including ants [21,24], invasive plants [58] and birds [59]. In a recent study using a combined data set including Chaetodontids, Pomacentrids and non-Scarine Labrids at a larger spatial scale, Mouillot et al. [60] also showed that nestedness was a major driver in reef fish community assembly. Similarly, Bender et al. [61] show that geographic isolation is important in structuring nestedness in coral reef communities at both taxonomic and functional levels.

Additional support for the importance of the longitudinal distance from the western diversity center in driving the patterns of diversity comes from our data, which show that reduction in species richness away from the coral triangle is not simply a function of habitat availability, although it does contribute to species richness. Longitude is relatively more important than reef area in determining community structure, suggesting that the Melanesian biodiversity gradient is more dependent on distance from the Coral Triangle than on habitat availability. This result contrasts with those of other authors [8,62] who found that habitat availability drove species distribution patterns at regional scales. While they found that longitude was also a significant factor in influencing structure, their analyses were on much larger spatial scales. The differences between our and previous results could have arisen by their inclusion of small, low area sea mounts and coral atolls in the remote Pacific and Indian oceans, which present important concentrations of shallow water habitat in the otherwise deep open ocean [60]. At local scales, such as those investigated in our study, part of the reason for a weak classic species area relationship may lie in the actual allocation of that area and reef morphology [62]. For large-scale studies that include ocean areas without suitable habitat, an additional degree of longitude may not necessarily yield additional coral habitat because coral islands are few and far between. This means that an increase in habitat area will result in greater species diversity than a similar increase in longitude, up weighting the species richness contribution of additional habitat patches. In our analysis, almost all degrees of longitude include habitable reefs, which better allows us to explore the relationship between the two. Parravicini et al. [63] also found that reef area and biogeographic province were the primary predictors of reef fish diversity at large spatial scales. Their analysis also resolved biophysical factors, such as surface water temperature and connectivity, which co-varied with diversity, which we did not test for in this study.

### Linking species traits to community ecology

A population’s persistence across a seascape is a result of individuals colonizing an area, and the subsequently the ability of those individuals to develop into a self-sustaining population. As most reef fish are site attached as adults, ecologically important connectivity must be mediated through a dispersive larval phase. Our results show that both dispersal and survival traits help explain the patterns of community structure we see along the Melanesian diversity gradient. At the Melanesian scale larval duration, an important dispersal trait, is positively correlated with geographic extent. Long larval duration has been a variable predictor of range size in the Indo-Pacific, with some researchers showing it to be a poor predictor except at the largest of spatial scales [e.g. 26], while other researchers have found positive relationships at the basin-wide scale for multiple species [64] and in damselfish, in Melanesia and in the Central Pacific [65,66]. These results suggest that spatial and taxonomic scales of the data could impact our expectations and interpretations with regards to the impact of larval duration. Supporting the importance of dispersal traits in this system, the family with the shortest larval durations (the Pomacentridae) is anti-nested, potentially indicating dispersal limitation among countries. We also saw a positive relationship between geographic extent and body size, which is associated with post-settlement survival. Larger fish are less subject to predation pressure and tend to have broader habitat preferences [26]. We expected to see a pattern of increased geographic extent in schooling fish, as those species have higher rates of anti-predator behavior and may have better survival [67]. Our data did not fully support that hypothesis, and more research linking behavioral traits, such as schooling behavior, to dispersal and range size is needed.

### Biogeographic patterns of diversity

Despite the overall structured nestedness of the communities there does exist geographic and taxonomic variation in the similarities among those communities. Both clustering analyses and decomposition of beta diversity indicate a faunal break between the islands of eastern Melanesia (Fiji, Tonga, and the Samoa) and the western islands. Although nestedness is evident for the entire dataset, when we examine patterns on a family level we find that the Pomacentridae are significantly anti-nested, meaning that populations on the periphery contain a unique assemblage of species. This is likely due to the large number of Pomacentridae species which are either endemic to Fiji, Tonga, and Samoa, or who have biogeographic affinities for central pacific reefs [68]. This also may be an artifact of recent alpha taxonomy work occurring within that region [69–71]. For example, of the 12 new species of Pomacentridae from this region that have been described since 2000, seven are from Fiji, Tonga or Samoa, four are from Papua New Guinea and/or the Solomon Islands, and one is broadly distributed across the region. The presence of so many endemic species in peripheral populations within the family that has the shortest dispersal rate, however, suggests that peripartric speciation could an important driver in developing unique ecological communities. Pomacentrids contain a suite of unique ecological traits (short durations, poor swimming ability, small body size) that could limit ecologically and evolutionary significant connectivity across the study system. This in turn suggests that the populations in Eastern Melanesia were probably independent from those in the Coral Triangle during quaternary glacial cycles of sea level change [7], providing both the time and geographic isolation necessary to develop unique communities. Other groups, such as Chaetodontids and Labrids, have longer larval duration and more robust swimming abilities. These traits likely maintained greater connectivity across Melanesia for these groups, resulting in their nestedness [7].

The communities in the Eastern end of Melanesia (Fiji, Tonga, Samoa) show patterns of differentiation that could have come about through a combination of multiple processes. The islands of eastern Melanesia may be drawing from a different species pool, such as the Polynesian or other Pacific Plate islands, than those in western Melanesia [72]. Additionally, some species may have evolved in the relative isolation of these peripheral populations (see above). Finally, and related, dispersal limitation has likely reduced the number of species that can disperse to and colonize the eastern islands. All three islands lie to the east of the Fiji plateau, an expanse of young oceanic crust [73] that is largely bereft of suitable habitat for coral reef fish. The oldest emergent parts of the islands date from the late Eocene to early Miocene [74–76], a time that matches periods of tip diversification for many of these reef fish fauna [77–80]. Past dispersal among now-submerged islands such as the Vitiaz Island Arc could have facilitated the initial reef fish colonization of eastern Melanesia, as is postulated for ants and birds [25, 81]. As those islands sank, dispersal became limited and led to isolation and subsequent speciation.

Data from both biogeography [7, 82] and genetics [15, 16] support the Center of Origin as a major driver of diversification within western Melanesia. The Center of Origin postulates that peripheral populations will be derived from ancestral populations found in the Coral Triangle. Here we find that the communities on the edge are also derived from those within the Coral Triangle. Layerd upon this are unique species found in the far Eastern Islands, especially in the Pomacentridae. The uniqueness of peripheral communities supports models of island evolution and isolation dating back to Wilson or earlier [22].

The faunal similarity of the Western Islands is reinforced by low levels of differentiation and strong clustering based on dissimilarity indices. The data we present here, along with others (e.g. [83–85]), show that populations in Papua New Guinea and the Solomon Islands contain numerous endemic species. These biogeographic affinities were also resolved by Kulbicki et al. [62] who found a soft edge for the “coral triangle” suggesting that the Melanesian province may extend past the Solomon Islands to Vanuatu, but not further west. Again we draw from David Starr Jordan’s speaking of the fish of Samoa [54] *“A few large species are confined to the islands of Polynesia and many small ones*… *seemed to have originated in Polynesia*.*”* Ultimately our data reinforce an emerging paradigm for the generation of biodiversity in the tropical Pacific that stresses a polygenic origin for biodiversity, with multiple processes acting in both synergistic and oppositional fashions. We do want to reiterate however that nestedness results are not causal. Nestedness itself it is not a driver, but a pattern of beta-diversity that suggests the observed faunal differences may be due to either differential colonization or extinction, without distinguishing between the two.

Our work highlights the usefulness of nuanced biogeographic analyses as applied to distributional data at a regional scale in helping prioritize regional conservation measures. Regardless of the mechanisms underpinning the community structure, clearly delineating the species richness and distribution across Melanesia provides us a useful tool for applying biogeography to conservation. Our results have two conservation implications at the regional level. The first is that the islands on either side of the Fiji plateau function as more tightly linked groups with those islands forming a natural unit for biodiversity management. Secondly, notwithstanding this structure driven by individual families, the countries in the east still largely form a nested subset of species found in the west. Conservation prioritization and regional management practices should incorporate these faunal relationships. Applying resources to protect the ecosystems, habitats and connections linking western Melanesian countries will provide benefit for all of Melanesia, while forming cohesive international conservation practices among Eastern Melanesia will help ensure protection for their unique species assemblages.

## Conclusions

We demonstrate that distance from the Coral Triangle drives patterns of community assembly despite differences in available habitat within countries, differences in species richness, and complex patterns of endemism in eastern Melanesia. These data provide useful information for regional level based conservation programs. At a more general level, our work demonstrates how large-scale, publically accessible databases can be mined for data to test ecological and conservation hypotheses. Moreover, diversity gradients occur over many spatial and taxonomic scales globally and while the research here is focused on tropical fish, it provides a useful methodological framework for researchers working in a diverse array of systems.

## Supporting information

S1 Fig Dendrograms of reef fish communities for five taxa (Balistidae, Chaetodontidae, Labridae, Monacanthidae, Pomacentridae) built on Jaccard distances and clustered using group averages. (DOCX)

S1 Table Results of nestedness analyses for each taxa, columns (reefs) ordered by richness in analysis. (DOCX)

S2 Table Results of nestedness analyses for each taxa, columns (reefs) ordered by longitude in analysis. (DOCX)

S3 Table An annotated list of 396 species distributions and trait data across seven countries in Melanesia. (XLSX)

S4 Table Citations used in generating S3. (DOCX)
