# Supplementary material for: Community assembly of coral reef fishes along the Melanesian biodiversity gradient
Source: PLoS One. 2017 Oct 25;12(10):e0186123. doi: 10.1371/journal.pone.0186123 (PMC5656311; doi:10.1371/journal.pone.0186123)
Supplement: S1 Fig — (DOCX) [file pone.0186123.s001.docx]

**Supplementary Information**

**S1 Fig. Dendrograms of reef fish communities for five taxa (Balistidae, Chaetodontidae, Labridae, Monacanthidae, Pomacentridae) built on Jaccard distances and clustered using group averages**. **A**: Total Jaccard distances. **B:** Jaccard distance due to species turnover/replacement (βrep). **C**: Jaccard distance due to richness differences (βrich). β diversity was partitioned into components following Carvalho et al. (2012).

**
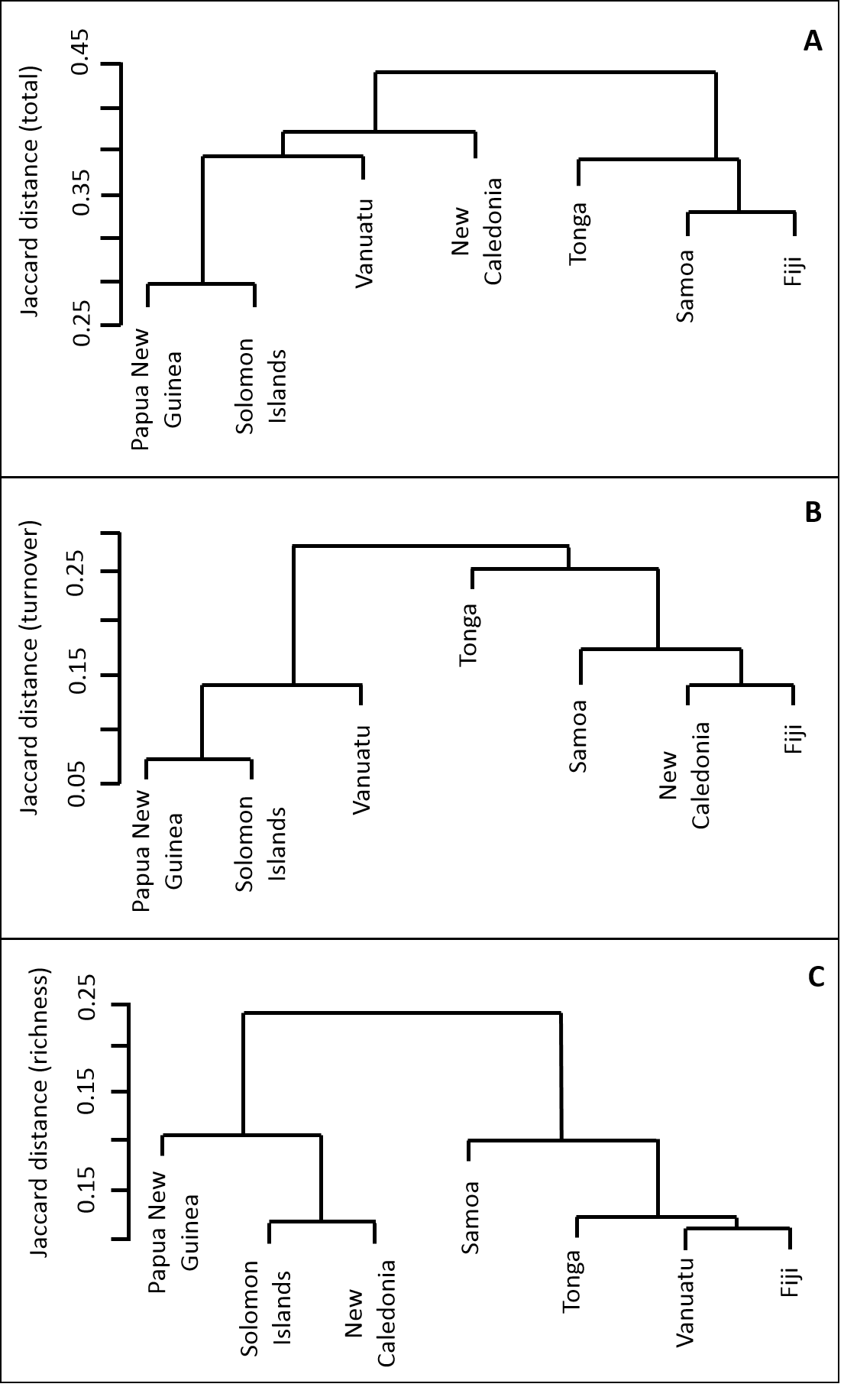
**
