# Supplementary material for: Community assembly of coral reef fishes along the Melanesian biodiversity gradient
Source: PLoS One. 2017 Oct 25;12(10):e0186123. doi: 10.1371/journal.pone.0186123 (PMC5656311; doi:10.1371/journal.pone.0186123)
Supplement: S1 Table — (DOCX) [file pone.0186123.s002.docx]

**S1 Table.** **Results of nestedness analyses for each taxa, columns (reefs) ordered by richness in analysis.** A metric of 100 indicates perfect nestedness. Significant Z-values (p<0.05) are indicated by bold. Number of species in each group indicated in parentheses.

|  | NODF | | | NODFc | | | NODFr | | |
| --- | --- | --- | --- | --- | --- | --- | --- | --- | --- |
|  | Metric | Z value | Pz(H0) | Metric | Z value | Pz(H0) | Metric | Z value | Pz(H0) |
| All species (396) | 73.09 | -0.45 | 0.325 | 84.34 | 0.57 | 0.284 | 73.09 | -0.41 | 0.342 |
| Balistidae (18) | 77.78 | 00.49 | 0.312 | 84.46 | -0.85 | 0.198 | 76.86 | -0.46 | 0.323 |
| Chaetodontidae (42) | 55.61 | 0.35 | 0.362 | 90.40 | 0.94 | 0.175 | 54.76 | 0.36 | 0.360 |
| Labridae (178) | 72.88 | -0.66 | 0.255 | 83.90 | 0.12 | 0.434 | 72.86 | -0.61 | 0.271 |
| Monacanthidae (15) | 81.50 | 0.60 | 0.273 | 86.75 | 0.52 | 0.301 | 80.44 | 0.55 | 0.290 |
| Pomacentridae (143) | **74.11** | **-1.83** | **0.034** | **85.90** | **-3.18** | **0.0007** | **74.09** | **-1.85** | **0.032** |
