# Supplementary material for: Community assembly of coral reef fishes along the Melanesian biodiversity gradient
Source: PLoS One. 2017 Oct 25;12(10):e0186123. doi: 10.1371/journal.pone.0186123 (PMC5656311; doi:10.1371/journal.pone.0186123)
Supplement: S2 Table — (DOCX) [file pone.0186123.s003.docx]

**S2 Table.** **Results of nestedness analyses for each taxa, columns (reefs) ordered by longitude in analysis**. A metric of 100 indicates perfect nestedness. Significant Z-values (p<0.05) are indicated by bold**.** Number of species in each group indicated in parentheses.

|  | NODF |  |  | NODFc |  |  | NODFr |  |  |
| --- | --- | --- | --- | --- | --- | --- | --- | --- | --- |
|  | Metric | Z value | Pz(H0) | Metric | Z vale | Pz(H0) | Metric | Zvalue | Pz(H0) |
| All species (396) | 73.09 | -0.44 | 0.329 | **73.87** | **4.31** | **<0.0001** | 73.09 | -0.41 | 0.342 |
| Balistidae (18) | 74.09 | -0.84 | 0.200 | **43.83** | **1.66** | **0.048** | 78.25 | -1.03 | 0.151 |
| Chaetodontidae (42) | 59.35 | -1.10 | 0.135 | 76.19 | -1.77 | 0.386 | 58.94 | -0.99 | 0.161 |
| Labridae (178) | 72.85 | -0.73 | 0.233 | **66.09** | **2.85** | **0.002** | 72.86 | -0.70 | 0.242 |
| Monacanthidae (15) | 75.09 | 0.57 | 0.284 | 48.34 | 0.40 | 0.345 | 80.44 | 0.50 | 0.308 |
| Pomacentridae (143) | **74.11** | **-1.81** | **0.035** | **85.90** | **-6.63** | **<0.0001** | **74.09** | **-1.91** | **0.028** |
