# Supplementary material for: Community assembly of coral reef fishes along the Melanesian biodiversity gradient
Source: PLoS One. 2017 Oct 25;12(10):e0186123. doi: 10.1371/journal.pone.0186123 (PMC5656311; doi:10.1371/journal.pone.0186123)
Supplement: S4 Table — (DOCX) [file pone.0186123.s005.docx]

**S4 Table.** **Citations used in generating S3.**

Allen GR. Damselfishes of the South Seas. TFH Publications. Neptune City, New Jersey USA. 1975.

Allen GR, Drew JA. A new species of damselfish (Pomacentrus: Pomacentridae) from Fiji and Tonga. Aqua, International Journal of Ichthyology 2012; 18: 171–80.

Allen GR, Randall JE. Three new species of wrasses (Labridae: Cirrhilabrus) from Papua New Guinea and the Solomon Islands. Revue Française D’aquariologie 1996; 23 (3-4): 101–11.

Allen GR, Randall JE, Carlson BA. *Cirrhilabrus marjorie*, a new wrasse (Pisces: Labridae) from Fiji. Aqua, Journal of Ichthyology and Aquatic Biology 2003; 7 (3): 113–18.

Allen GR. Damselfishes of the world. Melle, Germany: Mergus Publishers.; 1991.

Allen GR. Reef and shore fishes of Milne Bay Province, Papua New Guinea. In: Werner TB, Allen GR, editors. A rapid biodiversity assessment of the coral reefs of Milne Bay Province, Papua New Guinea. RAP Working Papers 11; Washington D.C.: Conservation International; 998. pp 39–49.

Allen GR, Drew J, Barber P. *Cirrhilabrus beauperryi,* a new wrasse (Pisces: Labridae) from Melanesia. Aqua, International Journal of Ichthyology 2008; 14 (3): 129-140.

Allen GR, Drew J, Fenner D. *Amphiprion pacificus,* a new species of anemonefish (Pomacentridae) from Fiji, Tonga, Samoa, and Wallis Island. Aquaculture 2010; 16: 129–38.

Allen GR, Emery A. A review of the Pomacentrid fishes of the genus Stegastes from the Indo-Pacific: With descriptions of two new species (Indo-Pacific Fishes, No3). Honolulu, Hawaii: Bishop Museum Press; 1985.

Allen GR, Erdmann MV. Reef fishes of the East Indies. Vol. 1. Honolulu, Hawaii: University of Hawaii Press; 2012.

Allen GR, Randall JE. Review of the Sharpnose Pufferfishes (subfamily Canthigasterinae) of the Indo-Pacific. Rec. Aust. Mus 1977; 30 (17): 475–517.

Allen GR, Steene RC, Allen M. A Guide to Angelfishes & Butterflyfishes. Odyssey Publishing, Hong Kong, China; 1998.

Almany GR, Berumen ML, Thorrold SR, Planes S, Jones GP. Local replenishment of coral reef fish populations in a marine reserve. Science 2007; 316 (5825): 742-4.

Bay LK, Choat JH, van Herwerden L, Robertson DR. High genetic diversities and complex genetic structure in an Indo-Pacific tropical reef fish (Chlorurus sordidus): evidence of an unstable evolutionary past? Marine Biology 2004; 144(4): 757-67.

Brothers EB, Williams DM, Sale PF. Length of larval life in twelve families of fishes at "One Tree Lagoon", Great Barrier Reef, Australia. Marine Biology 1983; 76(3): 319-324.

Brothers EB, Thresher RE. Pelagic duration, dispersal, and the distribution of Indo-Pacific coral reef fishes.  In: Reaka, ML, editor. The Ecology of Coral Reefs. Symposium Series for Undersea Research. Rockville: NOAA Undersea Research Program 3(1); 1985. pp 53-69.

Drew J, Allen GR, Kaufman L, Barber PH. Endemism and regional color and genetic differences in five putatively cosmopolitan reef fishes. Conservation Biology 2008; 22(4): 965-75.

Dudgeon CL, Gust N, Blair D. No apparent genetic basis to demographic differences in scarid fishes across continental shelf of the Great Barrier Reef. Marine Biology 2000; 137(5-6): 1059-1066.

Findley JS, Findley MT. Global, regional, and local patterns in species richness and abundance of butterflyfishes. Ecological Monographs 2001; 71(1): 69-91.

Gomon MF. 2006. A revision of the Labrid fish genus Bodianus with descriptions of seven new species. Records of the Australian Museum, Vol. 30; 2006.

Hoese DF, Bray DJ, Paxton JR, Allen GR. Fishes. In Beasley OL, Wells A (editors). Zoological catalogue of Australia. Volume 35. Australia: ABRS & CSIRO Publishing; 2006. Part 1, pp. xxiv 1-670; Part 2, pp. xxi 671-1472; Part 3, pp. xxi 1473-2178.

Hunnam P, Jenkins A, Kile N, Shearman P. Marine resource management and conservation planning. Bismarck-Solomon Seas ecoregion: Papua New Guinea, Solomon Islands. WWF; 2001.

Kailola PJ. The fishes of Papua New Guinea: A revised and annotated checklist. Vol. II. Scorpaenidae to Callionymidae. Research Section, Department of Fisheries and Marine Resources, Port Moresby: Research Bulletin 41; 1987.

Kobayashi D, Friedlander A, Grimes C, Nichols R, Zgliczynski B. Bumphead parrotfish (Bolbometopon muricatum) status review. NOAA Technical Memorandum NMFS-PIFSC-26. NOAA; 2011.

Kulbicki MJ, Randall E, Rivaton J. Checklist of the fishes of the Chesterfield Islands (Coral Sea). Micronesica 1994; 27 (1/2): 1–43.

Lieske W, Myers R. Coral reef fishes. Indo-Pacific & Caribbean including the Red Sea. Collins Pocket Guide. New York: HarperCollins Publishers; 1994.

Luiz OJ, Allen AP, Robertson DR, Floeter SR, Kulbicki M, Vigliola L, et al. Adult and larval traits as determinants of geographic range size among tropical reef fishes. Proceedings of the National Academy of Sciences of the United States of America 2013; 110(41): 16498-502.

McCormack G. Cook Islands biodiversity and natural heritage database. Sent by Gerald McCormack as RTF document in May 2000 for use in FishBase.

Myers RF. Micronesian reef fishes: A practical guide to the identification of the coral reef fishes of the tropical central and western pacific. Coral Graphics; 1991.

Nakamura Y, Shibuno T, Yamaoka K. Relationship between pelagic larval duration and abundance of tropical fishes on temperate coasts of Japan. Journal of Fish Biology 2012; 80(2): 346-357.

Parenti P, and Randall JE. An annotated checklist of the species of the Labroid fish families Labridae and Scaridae. Ichthyological Bulletin of the JLB Smith Institute of Ichthyology 2000; 68: 1–97.

Parenti P, and Randall JE. Checklist of the Species of the Families Labridae and Scaridae: An Update. Smithiana Bulletin 2011; 13: 29-44.

Pyle RL, Earle JL, Greene BD. Five new species of the damselfish genus Chromis (Perciformes : Labroidei : Pomacentridae) from deep coral reefs in the tropical western Pacific. Zootaxa 2008(1671): 3-31.

Randall JE, Allen GR, Steene RC. Fishes of the Great Barrier Reef and Coral Sea. Bathurst: Crawford House; 1990.

Randall JE, Pyle RM. Cirrhilabrus scottorum, a new Labrid fish from the south Pacific Ocean. Revue Française D’aquariologie 1988; 15 (4): 113–18.

Randall JE. A revision of the Labrid fish genus Anampses. Micronesica. 1972; 8 (1): 151–95.

Randall JE. Reef and shore fishes of the South Pacific: New Caledonia to Tahiti and the Pitcairn Islands. Vol. 1. Honolulu: University of Hawaii Press; 2005.

Randall JE, Allen GR, Steene RC. Fishes of the Great Barrier Reef and Coral Sea. Honolulu: University of Hawaii Press; 1997.

Randall JE, Anderson RC. Annotated checklist of the epipelagic and shore fishes of the Maldives Islands. JLB Smith Institute of Ichthyology, Ichtyhological Bulletin 1993 59: 1-45.

Randall JE, and J. Howard Choat. Two new parrotfishes of the genus Scarus from the central and south Pacific, with further examples of sexual dichromatism. Zoological Journal of the Linnean Society 1980; 70 (4): 383–419.

Randall JE, Justine JL. The triggerfish Abalistes filamentosus from New Caledonia, a first record for the South Pacific. Cybium 2008;32(2):183-4.

Randall JE. A review of the labrid fishes of the genus Cirrhilabrus from Japan,

Taiwan and the Mariana Islands, with descriptions of two new species. Micronesica 1992; 25(1):99-121.

Randall JE, King DR. Halichoeres zulu, a new Labrid fish from South Africa. Smithiana Bulletin 2010; 11: 17-23.

Randall JE, Williams JT, Smith DG, Kulbicki M, Mou Tham G., Labrosse P, Kronen M, Clua E, Mann BS. Checklist of the shore and epipegagic [sic] fishes of Tonga. Atoll Research Bulletin No. 502. Washington DC: Smithsonian Inst. 2003.

Thresher RE, Colin PL, Bell LJ. Planktonic duration, distribution, and population-structure of western and central Pacific damselfishes (Pomacentridae). Copeia 1989(2): 420-34.

Seeto J, Baldwin WJ. A checklist of the fishes of Fiji and a bibliography of Fijian fish. The University of the South Pacific Marine Studies Technical Report 220; 2010.

Shea S, Liu M. 2010. Choerodon margaritiferus. In: IUCN 2013. IUCN Red List of

Threatened Species. Version 2013.1. <www.iucnredlist.org>. Downloaded on 23 September 2013.

Stobutzki IC. Interspecific variation in sustained swimming ability of late pelagic stage reef fish from two families (Pomacentridae and Chaetodontidae). Coral Reefs 1998;17(2):111-9.

Thaman RR, Puia T, Tongabaea W, Namona A, Fong T. Marine biodiversity and ethnobiodiversity of Bellona (Mungiki) island, Solomon Islands. Singapore Journal of Tropical Geography 2010; 31 (1): 70–84.

Wantiez L, HarmelinVivien M, Kulbicki M. Spatial and temporal variation in a soft-bottom fish assemblage in St Vincent Bay, New Caledonia. Marine Biology 1996; 125(4): 801-12.

Wass RC. An annotated checklist of the fishes of Samoa. US Department of Commerce, NOAA, National Marine Fisheries Service 1984.

Wellington GM, Victor BC. Planktonic larval duration of one hundred species of Pacific and Atlantic damselfishes (Pomacentridae). Marine Biology 1989; 101(4): 557-567.

Westneat, M. W. 2001. Labridae. Wrasses, Hogfishes, Razorfishes, Corises, Tuskfishes. FAO Species Identification Guide for Fishery Purposes. The Living Marine Resources of the Western Central Pacific 6: 3381–3467.

Wilson DT, McCormick MI. Microstructure of settlement-marks in the otoliths of tropical reef fishes. Marine Biology 1999; 134(1): 29-41.

Victor BC. Duration of the planktonic larval stage of one hundred species of Pacific and Atlantic wrasses (family Labridae). Marine Biology 1986; 90(3): 317-326.
